# Creating Standardized Tools for the Pharmacist-Led Assessment and Pharmacologic Management of Adult Canadians Wishing to Quit Smoking: A Consensus-Based Approach

**DOI:** 10.3390/pharmacy9020080

**Published:** 2021-04-14

**Authors:** Kristi Butt, Nardine Nakhla

**Affiliations:** School of Pharmacy, University of Waterloo, Kitchener, ON N2G 1C5, Canada; kmbutt@uwaterloo.ca

**Keywords:** non-prescription medicines, over the counter, self-care, community pharmacy, health promotion, health prevention, harm reduction, smoking cessation, tobacco cessation, harmonized pharmacist-led care

## Abstract

Tobacco use continues to be recognized as the single most preventable cause of death worldwide. As the gatekeepers of and experts on pharmacotherapy, pharmacists play a vital role in facilitating smoking cessation. While existing frameworks have enabled pharmacists to provide smoking cessation services in Canada for many years, the way in which they are delivered vary considerably across the nation. The purpose of this initiative was to create standardized tools for the pharmacists providing cessation services to ensure all Canadians wishing to stop smoking have equal access to consistent, evidence-based care. An iterative process using repeated rounds of voting was employed to establish consensus among key opinion leaders on the most important items to include in tools for the pharmacist-led assessment and pharmacologic management of Canadian adults wishing to stop smoking. The results were used to create eight standardized documents for national use by pharmacists: a readiness to quit assessment tool, a patient consent form, a patient assessment form for past users of tobacco and/or tobacco-like products, a patient assessment form for current users of tobacco and/or tobacco-like products, a treatment algorithm, a treatment plan summary form, a prescribing documentation form, and a follow-up & monitoring documentation form. Although not described in detail in these documents, other strategies for smoking cessation (e.g., non-pharmacologic strategies (including quitting “cold turkey” and behavioural interventions), harm reduction strategies, etc.) should be considered when pharmacotherapy is inappropriate or undesired; care should be individualized based on a patient’s previous experiences and current motivation. No single approach to treatment is endorsed by the authors. The consensus-based approach described here provides a suggested framework for harmonizing the pharmacist-led management of other ailments to optimize patient care.

## 1. Introduction

Smoking causes an estimated 45,000 deaths in Canada each year, representing approximately 18% of all annual deaths [1]. Nearly all deaths attributed to smoking in Canada are related to cancer, cardiovascular disease or respiratory disease, with cancer being the most prevalent [1]. Tobacco use more generally continues to be recognized as the single most preventable cause of premature death in Canada and globally [2,3]. According to the 2017 Canadian Tobacco, Alcohol and Drugs Survey, 18% of Canadians 15 years of age and older reported using at least one tobacco product in the past 30 days; an increase from 15% in 2015 [4]. On 31 May 2018 the Government of Canada announced Canada’s Tobacco Strategy, which aims to reduce tobacco use to less than 5% by 2035 [2]. 

Approximately half of Canadians who smoke make at least one quit attempt per year [5]. Research shows that smoking cessation is more likely with advice from a healthcare professional (HCP) and when behavioural support and pharmacotherapy are used in combination [6,7,8]. As the gatekeepers of and experts on pharmacotherapy, pharmacists will play a pivotal role in helping achieve the ambitious goal of less than 5% tobacco use by 2035; particularly since a first-line treatment option for smoking cessation, nicotine replacement therapy (NRT), is available without a prescription in Canada, meaning a pharmacist may be the only HCP a patient sees before purchasing and initiating therapy to quit smoking. 

Existing frameworks have enabled pharmacists to provide smoking cessation services in Canada for many years. However, the way in which these services are delivered vary considerably according to province or territory [9]. For example, pharmacists in Ontario and Saskatchewan have required protocol(s) or algorithm(s) for smoking cessation counselling and prescribing, whereas pharmacists in other provinces and territories do not [9]. This suggests patients attempting to quit smoking may not receive the same type and quality of care from pharmacists across Canada. The desire for a formal approach to providing smoking cessation services was identified as an important theme in discussions with key stakeholders at the National Pharmacist-led Smoking Cessation Symposium held on 12 June 2018 in Ottawa, ON, Canada [9]. 

The aim of this work was to establish consensus among key opinion leaders (KOLs) on the essential items to include in tools for the pharmacist-led assessment and pharmacologic management of patients wishing to quit smoking. To evaluate the feasibility and effectiveness of the proposed approach in reaching consensus, only items on tools currently used for providing smoking cessation care to adults in Canada were considered. Establishing consensus on tools for providing care to special patient populations (e.g., adolescents) and recommendations concerning more controversial treatment approaches (e.g., using e-cigarettes as a smoking cessation aid) was beyond the scope of this work. E-cigarettes in particular were omitted as there were ongoing concerns surrounding the standardization and regulation of these products in Canada and no formal tools on their use in smoking cessation had been published at the time of this work. 

Results were used to create standardized documents for pharmacists providing smoking cessation services with the goal of ensuring all adults in Canada wishing to stop smoking have equal access to consistent, evidence-based care. Although the pharmacologic management of patients is highlighted in these documents, the patient assessment form guides pharmacists in exploring the details of past quit attempts (e.g., strategies tried, reason(s) for stopping), such that management can be individualized based on a patient’s previous experiences and current preferences. No single approach to treatment is endorsed by the authors. If pharmacotherapy is inappropriate or undesired, other approaches to management (e.g., non-pharmacologic strategies (including quitting “cold turkey” and behavioural interventions), harm reduction strategies, etc.) should be considered. 

The consensus-based approach used to create standardized tools also provides a suggested framework for harmonizing the pharmacist-led management of other ailments. 

## 2. Materials and Methods

### 2.1. Pre-Voting

Existing tools used by pharmacists providing smoking cessation services in Canada were gathered from KOLs and stakeholders who attended the 2018 National Pharmacist-led Smoking Cessation Symposium. An independent online search was also conducted by the research team to identify other existing tools. Items from tools collected were compiled.

### 2.2. Recruitment of Participants

Pharmacists licensed in Canada with expertise in smoking cessation were identified through Pharmacists for a Smoke-Free Canada (http://psfcnetwork.com, accessed on 23 August 2019) or their attendance at the 2018 National Pharmacist-led Smoking Cessation Symposium. The ability to read, write, and understand English was also a requirement for participation, as translation services were not available. At least one eligible pharmacist from each Canadian province and territory were invited to participate via email. 

### 2.3. Online Pilot Questionnaire 1 

Qualtrics^®^ (Seattle, WA, USA) was used to create an online pilot questionnaire on items compiled from existing tools used by pharmacists providing smoking cessation services. Participants were asked to “agree” or “disagree” with the inclusion of each item on standardized tools for national use. For each item, participants were able to provide written feedback on their decision (e.g., arguments to justify their viewpoints). Consensus was met if ≥80% agreed or disagreed with an item’s inclusion. Eighty percent has been suggested as the minimum percentage of experts that must agree on an item to achieve content validity when there are at least ten experts participating in consensus development [10,11]. Participants were also able to propose new items for questionnaire 2. Results were anonymous. Consent was implied by participants choosing to complete the questionnaire. 

### 2.4. Online Pilot Questionnaire 2

After eliminating redundancies, items not achieving consensus on the first questionnaire were included on a second online pilot questionnaire created using Qualtrics^®^. New items proposed by participants were also included. Participants were provided with results and relevant comments from questionnaire 1 to consider in their decision-making for items on questionnaire 2. Again, participants were asked to “agree” or “disagree” with the inclusion of each item in standardized tools for national use. For each item, participants were able to provide written feedback on their decision. Consensus was met if ≥80% agreed or disagreed with an item’s inclusion. Participants were also able to propose new items for live voting. Results were anonymous. Consent was implied by participants choosing to complete the questionnaire.

### 2.5. Live Voting

After eliminating any remaining redundancies, items not achieving consensus on the second questionnaire were included for live voting at the 2019 National Pharmacist-led Smoking Cessation Symposium held on 19 October in Kitchener, Ontario, Canada. New items proposed by participants were also included. Participants were provided with results and relevant comments from questionnaire 2 to consider in their voting. For each item, participants were asked to “agree” or “disagree” with the inclusion of each item in standardized tools for national use verbally or by using a show of hands. Again, consensus was met if ≥80% agreed or disagreed with an item’s inclusion. Items with <80% agreement or disagreement were discussed until participants reached consensus. Results were not anonymous. Consent was implied by participants choosing to vote. Live voting received ethics clearance through a University of Waterloo Research Ethics Committee (ORE#41281). 

## 3. Results

Thirteen pharmacists with expertise in smoking cessation completed the two online questionnaires and twelve participated in the live voting session. Participants included at least one representative from each Canadian province and territory, except Quebec. Other characteristics of participants are described in Table 1. Four hundred and twelve items compiled from existing tools used by pharmacists when providing smoking cessation services were included on questionnaire 1. Of those items, participants agreed that 283 should be included on standardized forms for national use and none should be omitted. Consensus was not met for 129 items on questionnaire 1. These items, along with new items proposed on questionnaire 1, were included on questionnaire 2. Of the 172 items on questionnaire 2, participants agreed that 50 should be included on standardized forms for national use and 8 should be omitted. Consensus was not met for 114 items on questionnaire 2. Redundancies in items not achieving consensus were eliminated and the remaining 47 items were included for live voting. With facilitated discussion among participants, consensus was met on all items included for voting. 

Examples of items that achieved and failed to achieve consensus in the two questionnaires and live voting session are provided in Table 2. Results from the online questionnaires and live voting session were used to assemble standardized tools for national use by pharmacists providing smoking cessation services. If ≥ 80% of participants agreed with the inclusion of an item, it was included in the tool. If ≥ 80% disagreed with the inclusion of an item, it was omitted from the tool. Once assembled, tools were sent to participants for final feedback on content, organization, and formatting. Eight standardized documents were created using this consensus-based approach, including a readiness to quit assessment tool, patient assessment form for current users of tobacco and/or tobacco-like products, treatment algorithm, and prescribing documentation form. Other documents include a patient consent form, a patient assessment form of past users of tobacco and/or tobacco-like products, a treatment plan summary form, and a follow-up & monitoring documentation form (see Supplementary Materials for information on accessing full toolkit).

## 4. Discussion

A consensus-based approach was used to identify essential items for inclusion in standardized tools for the pharmacist-led assessment and pharmacologic management of adults wishing to quit smoking in Canada. Similar approaches, like the Modified Delphi—an iterative process using repeated rounds of voting to establish consensus among experts—are often used in health fields to develop guidelines and policies when evidence is limited and/or opinion is important [10]. To our knowledge, this is the first time a consensus-based approach has been used to develop standardized tools for pharmacist-led smoking cessation services in Canada. 

Tobacco use is the leading cause of preventable premature disease in Canada [2]. Furthermore, smoking is thought to be responsible for approximately 18% of all deaths annually [1]. The economic burden of tobacco use in Canada is also significant, producing an estimated $6.5 billion CAD in direct healthcare costs and $9.5 billion CAD in indirect costs annually [1]. However, concerns surrounding tobacco use are not unique to Canada. The World Health Organization (WHO) continues to identify tobacco use as “the biggest single preventable cause of death” globally, with more than seven million deaths each year from direct tobacco use and an additional 1.2 million from second-hand smoke [3,12]. In 2012, the total cost of smoking globally, including health expenditures and lost productivity, was $1436 billion USD, which is equivalent to almost 2% of the world’s annual gross domestic product [13]. 

The International Self-Care Foundation recommends “not smoking or quitting smoking” under Pillar 5 (risk avoidance or mitigation) of their seven-pillar framework for self-care. This recommendation is on the basis that “avoiding high-risk behaviours, particularly tobacco smoking and alcohol intake, have been shown to significantly reduce preventable mortality and reduce all-cause mortality” [14]. It is also highlighted under Pillar 5 that “behaviours that reduce health risks are often some of the most achievable self-care practices” and that “while quitting smoking is difficult, the benefit is significant, immediate and there are a range of options to aid people” [14]. While many adult smokers report they want or intend to quit, WHO reports only about 30% of the world’s population have access to appropriate tobacco cessation services [3]. 

NRT, an effective pharmacologic option for smoking cessation, is available from a pharmacy in Canada and many other countries (e.g., Kenya, Brazil, Thailand, Switzerland, Saudi Arabia, Japan) without a prescription [3]. This means a pharmacist may be the only HCP a patient sees before purchasing and initiating therapy for quitting smoking. Meta-analysis suggests pharmacy-delivered interventions for smoking cessation, including behavioural support and/or NRT, can be both clinically- and cost-effective [15]. Ensuring pharmacists are equipped with standardized evidence-based tools to facilitate clinical decision-making and documentation when providing smoking cessation services will harmonize care and presumably improve quit rates and promote public health. 

With this work, consensus was established on the essential items to include in tools for the pharmacist-led assessment and pharmacologic management of adults wishing to quit smoking in Canada. This information was used to create a toolkit of eight standardized documents, including a readiness to quit assessment tool, patient assessment form for current users of tobacco and/or tobacco-like products, treatment algorithm, and prescribing documentation form. Other documents include a patient consent form, a patient assessment form of past users of tobacco and/or tobacco-like products, a treatment plan summary form, and a follow-up & monitoring documentation form (see Supplementary Materials for information on accessing full toolkit). Although not described in detail in these documents, other strategies for smoking cessation (e.g., non-pharmacologic strategies (including quitting “cold turkey” and behavioural interventions), harm reduction strategies, etc.) should be considered when pharmacotherapy is inappropriate or undesired. The patient assessment form facilitates pharmacists in providing care that is individualized based on a patient’s previous experiences and current motivation. The authors do not endorse any single approach to treatment. 

The development of standardized tools for the assessment and pharmacologic management of patients wishing to quit smoking represents a critical step in harmonizing pharmacist-led smoking cessation in Canada and ensuring all residents have equal access to consistent, evidence-based care. This work also provides a suggested framework for harmonizing the pharmacist-led management of other ailments. Possible limitations of this work include the small number of participants involved in establishing consensus, not having representation from Quebec, and the lack of anonymity during in-person voting, which may have influenced results. 

Future efforts should focus on establishing consensus on standardized tools for providing smoking cessation services to special patient populations and recommendations concerning non-pharmacologic or more controversial approaches to treatment (e.g., the use of e-cigarettes). A process for ensuring standardized tools are updated regularly to reflect current evidence and best practices should also be developed and implemented. Other efforts should include piloting the standardized tools in practice and overcoming regulatory, infrastructural, and financial barriers to nationwide implementation. Consideration should also be given to developing a national smoking cessation curriculum for entry-to-practice and continuing education programs to ensure consistency in the training and knowledge of pharmacists licensed in Canada. With the tobacco epidemic being one of the biggest public health threats the world has faced [12], other countries lacking a harmonized framework for providing pharmacist-led smoking cessation services may also benefit from creating standardized tools for this purpose using the consensus-based approach described here.

## Figures and Tables

**Table 1:** Participant characteristics.

		Number of Participants (%)
Gender	Male	7 (54%)
	Female	6 (46%)
Age	20–30 years	1 (8%)
	31–40 years	4 (31%)
	41–50 years	5 (38%)
	51–60 years	1 (8%)
	61–70 years	2 (15%)
Country of undergraduate pharmacy degree	Canada	12 (92%)
	Outside of Canada	1 (8%)
Years licensed to practice pharmacy in Canada	0–10 years	4 (31%)
	11–20 years	5 (38%)
	21–30 years	1 (8%)
	31–40 years	2 (15%)
	41–50 years	1 (8%)
Province and/or territory currently licensed to practice pharmacy in	Alberta	1 (8%)
	British Columbia	2 (15%)
	Manitoba	2 (15%)
	New Brunswick	1 (8%)
	Newfoundland & Labrador	1 (8%)
	Northwest Territories	1 (8%)
	Nova Scotia	1 (8%)
	Nunavut	2 (15%)
	Ontario	4 (31%)
	Prince Edward Island	1 (8%)
	Quebec	0 (0%)
	Saskatchewan	1 (8%)
	Yukon	1 (8%)
Primary practice setting	Community pharmacy	5 (38%)
	Hospital pharmacy	1 (8%)
	Ambulatory care (e.g., family health team)	3 (23%)
	Education	2 (15%)
	Provincial advocacy association	1 (8%)
	Administration	1 (8%)
	**Select responses**	
Qualifications and/or experience with pharmacist-led smoking cessation	● Certification(s) through the: ○ Canadian Network for Respiratory Care ○ Centre for Addiction and Mental Health (TEACH Program) ○ Canadian Pharmacists Association (QUIT Program) ○ Clinical Tobacco Intervention Program ○ Ontario Pharmacists Association ● Consultant for the Government of Nunavut’s Tobacco Reduction Program ● Lead the Nova Scotia Let’s Quit Demonstration Project ● Developer and lead of the CATALYST Program	

**Table 2:** Examples of items that achieved and failed to achieve consensus in questionnaires and live voting.

		Items Achieving Consensus		Items Failing to Achieve Consensus for Inclusion/Exclusion
		For Inclusion	For Exclusion	
Readiness to quit assessment tool	Questionnaire 1	“Have you used any form of tobacco and/or tobacco-like products in the past 30 days? Yes or no”	N/A (no items achieved consensus for exlusion)	“On a scale of 1 (not important) to 10 (very important), how important is it for you to change your tobacco and/or tobacco-like product use now?”
	Questionnaire 2	A statement congratulating never or previous users of tobacco and/or tobacco-like products on their healthy choice	“How would you like to quit: cold turkey or with assistance?”	
	Live voting	“On a scale of 1 (not important) to 10 (very important), how important is it for you to change your tobacco and/or tobacco-like product use now?”	“Do you have insurance coverage for smoking cessation aids and/or services?”	N/A (consensus was met on all items)
Patient assessment: current users of tobacco and/or tobacco-like products	Questionnaire 1	“Do you drink caffeinated beverages (e.g., coffee, tea, pop) when you use tobacco and/or tobacco-like products? Yes (number of drinks per day: ___) or no.”	N/A (no items achieved consensus for exlusion)	“Are chronic medical conditions (if any) appropriately treated and controlled? If yes, proceed to treatment; if no, consult or refer patient to phyisician or nurse practitioner.”
	Questionnaire 2	“How many times have you tried to quit?”		
	Live voting	“What do you like about using tobacco/tobacco-like products?”	“Are chronic medical conditions (if any) appropriately treated and controlled? If yes, proceed to treatment; if no, consult or refer patient to phyisician or nurse practitioner.”	N/A (consensus was met on all items)
Treatment algorithm	Questionnaire 1	First-line pharmacotherapy options: NRT, varenicline, bupropion SR	N/A (no items achieved consensus for exlusion)	Third-line pharmacotherapy options: clonidine, cytisine, nortriptyline
	Questionnaire 2	Severe hepatic impairment as a contraindication for bupropion SR therapy		
	Live voting	Third-line pharmacotherapy options: clonidine, cytisine, nortriptyline	A statement about the possible emergence of neuropsychiatric symptoms in patients being treated with varenicline	N/A (consensus was met on all items)
Prescribing documentation form	Questionnaire 1	Name and strength of medication	N/A (no items achieved consensus for exlusion)	Rationale for prescription/relevant patient information
	Questionnaire 2	N/A (no items achieved consensus for inclusion)	Category of prescribing: Schedule I, II, III, or Unscheduled drug	
	Live voting	Rationale for prescribing, including desired or expected outcomes	License or registration number of primary care provider notified	N/A (consensus was met on all items)

## Data Availability

The data presented in this study are available on request from the corresponding author. The data are not publicly available to uphold privacy as stipulated in the research and ethics application.

## Supplementary Materials

The following are available online at https://www.mdpi.com/article/10.3390/pharmacy9020080/s1. For information on accessing the full toolkit, please contact Nardine Nakhla (nnakhla@uwaterloo.ca).
