# Supplementary material for: Creating Standardized Tools for the Pharmacist-Led Assessment and Pharmacologic Management of Adult Canadians Wishing to Quit Smoking: A Consensus-Based Approach
Source: Pharmacy (Basel). 2021 Apr 14;9(2):80. doi: 10.3390/pharmacy9020080 (PMC8167644; doi:10.3390/pharmacy9020080)
Supplement: Supplementary file 1 [file pharmacy-09-00080-s001.pdf]

READINESS TO QUIT ASSESSMENT

Name: ____________________

Date of birth: ____________________

Health card number (*or equivalent*):

____

Gender (*if required/relevant*): ____________________

Address: ____________________

____

Telephone number: ____________________

Primary care provider: ____________________

Do you have private drug coverage?

☐ Yes ☐ No

If yes:

Insurance provider: ____________________

Carrier: ____________________

Contract number: ____________________

Client ID: ____________________

Have you used any form of tobacco (*e.g., cigarettes, cigars, smokeless tobacco*) and/or tobacco-like products *(e.g., e-cigarettes)* in the past 30 days?

☐ Yes → *Please skip ahead to question 2*

☐ No → **Are you a previous user of tobacco and/or tobacco-like products?**

☐ Yes → **Are you interested in support from the pharmacist to remain tobacco/tobacco-like product free?**

☐ Yes

☐ No

☐ No

For pharmacist use only

If yes, ask patient to complete the patient assessment form for past users of tobacco and/or tobacco-like products (page 4)

Congratulations on your healthy choice! You may return this form to pharmacy staff without completing any further questions.

Are you willing to briefly discuss your use of tobacco and/or tobacco-like products?

☐ Yes → *Please complete the rest of this form*

☐ No → *If you would like to discuss this in the future, please contact your pharmacist. You may return this form to pharmacy staff without completing any further questions.*

How <u>important</u> is it for you to change your tobacco and/or tobacco-like product use now?

1 (not important) — 5 — 10 (very important)

How <u>confident</u> are you that you can change your use of tobacco and/or tobacco-like products?

1 (not important) — 5 — 10 (very important)

For pharmacist use only

If low, enhance readiness by exploring:

- *Rewards of tobacco/tobacco-like product use*
- *Risks of tobacco/tobacco-like product use*
- *Reflective listening (page 14) Roadblocks to quitting*
- *Different change behavioural goals (e.g., quitting vs. reducing)*

Repeat as necessary until importance and confidence are high

This toolbox may not be reproduced or distributed, in whole or in part, without prior permission. For more information, please contact the NPLSC lead, Nardine Nakhla, at nnakhla@uwaterloo.ca

How ready are you to change your tobacco and/or tobacco-like product use right now?

1 (not important)				5					10 (very important)

As your pharmacist, I strongly advise you to quit or reduce your use of tobacco and/or tobacco-like products, as it is the most important thing you can do for your health. I can help you do this.

Are you interested or willing to make a change in your tobacco use and/or use of tobacco-like products in the next 30 days?

- ☐ Yes
- ☐ No

For pharmacist use only

If yes, create a plan for change with the patient or schedule a visit to do so

If no, encourage patient to contact you when they are ready and/or offer self-help materials

For pharmacist use only

Comments *(pharmacist rating of importance of change/treatment at this time):*

Pharmacist: ______________________________

Date: ______________________________

This toolbox may not be reproduced or distributed, in whole or in part, without prior permission. For more information, please contact the NPLSC lead, Nardine Nakhla, at nnakhla@uwaterloo.ca

PATIENT ASSESSMENT:

CURRENT USERS OF TOBACCO AND/OR TOBACCO-LIKE PRODUCTS

Date of completion (*dd/mm/yy*): ____________________

Appointment location (*note: when possible, the first meeting should be in-person at the pharmacy*):

- ☐ In-person
- ☐ Telephone
- ☐ Video-conferencing
- ☐ Other *(please specify)*: ____________________

Name: ____________________

Date of birth: ____________________

Health card number (*or equivalent*):

____

Gender (*if required/relevant*): ____________________

Address: ____________________

____

Telephone number: ____________________

Primary care provider: ____________________

Medical History

Height: ____________________

Weight: ____________________

Allergies or intolerances:

Cardiovascular

- ☐ Heart attack within previous 14 days
- ☐ Angina
- ☐ Stroke
- ☐ High blood pressure
- ☐ Arrythmia
- ☐ Heart failure
- ☐ Family history of heart disease
- ☐ Other heart-related conditions (*specify*): ____________________

This toolbox may not be reproduced or distributed, in whole or in part, without prior permission. For more information, please contact the NPLSC lead, Nardine Nakhla, at nnakhla@uwaterloo.ca

Respiratory

- ☐ Asthma
- ☐ COPD
- ☐ Other lung-related conditions (*specify*): ______________________________

Neurologic

- ☐ History of seizures

Mental Health

- ☐ Depression
- ☐ Anxiety
- ☐ Bipolar
- ☐ Schizophrenia
- ☐ Substance and/or alcohol use disorder
- ☐ Eating disorder

If you checked any of the mental health issues listed above, have your medications changed or have you been hospitalized recently?

- ☐ Yes
- ☐ No

Other

- ☐ Cancer
- ☐ Gastrointestinal conditions (*e.g., heartburn, ulcers*)
- ☐ Unexplained weight loss
- ☐ Diabetes
- ☐ High cholesterol
- ☐ Liver disease
- ☐ Kidney disease (*CrCl* (*if known*): ______________)

If applicable:

- ☐ Pregnancy
- ☐ Breastfeeding

Smoking-related symptoms

- ☐ Cough
- ☐ Wheezing
- ☐ Shortness of breath

Conditions not previously indicated: ______________________________

This toolbox may not be reproduced or distributed, in whole or in part, without prior permission. For more information, please contact the NPLSC lead, Nardine Nakhla, at nnakhla@uwaterloo.ca

Medication History

☐ Completed below

☐ Best Possible Medication History or Patient Medication Profile attached

Schedule 1, 2, and 3 drugs, blood products, and natural health products currently being taken by the patient, as well as any recent (*within the last 6 months*) vaccines:

Product Name	Strength	Directions for Use	Indication

Other health products, aids, or devices being used by the patient that may affect the pharmacist's decision-making:

Review of Tobacco Use and/or Use of Tobacco-like Products

☐ Daily tobacco and/or tobacco-like product user

☐ Occasional tobacco and/or tobacco-like product user

For tobacco and/or tobacco-like products being used, indicate:

Type	Amount	Years of Use

Note: for cigarette users, # of pack-years = years smoked x packs per day

How soon after waking do you use tobacco and/or tobacco-like products?

☐ Within 30 minutes

☐ After 30 minutes

Where do you use tobacco and/or tobacco-like products most often? ______________________________

Do you find it difficult to refrain from using tobacco and/or tobacco-like products where it is forbidden (*e.g., in church, at the library, in cinema*)?

☐ Yes

☐ No

Do you use tobacco and/or tobacco-like products even if you are so ill you are in bed most of the day?

☐ Yes

☐ No

This toolbox may not be reproduced or distributed, in whole or in part, without prior permission. For more information, please contact the NPLSC lead, Nardine Nakhla, at nnakhla@uwaterloo.ca

If you chew tobacco, do you intentionally swallow the juices?

- ☐ Yes
- ☐ No

Do you drink alcohol when you use tobacco and/or tobacco-like products?

- ☐ Yes → *Number of drinks per day:* ______________
- ☐ No

Do you drink caffeinated beverages (*e.g., coffee, tea, pop*) when you use tobacco and/or tobacco-like products?

- ☐ Yes → *Number of drinks per day:* ______________
- ☐ No

With whom do you use tobacco and/or tobacco-like products?

- ☐ Alone
- ☐ Socially
- ☐ Both (*alone and socially*)

How many tobacco users reside in your home? ________

Are you a source of second-hand smoke or vapour for family and friends?

- ☐ Yes
- ☐ No

Are you currently under the care of your primary care provider (*e.g., family physician, nurse practitioner*) for quitting tobacco and/or tobacco-like products?

- ☐ Yes
- ☐ No

Previous Quit Attempts

Have you tried to quit using tobacco and/or tobacco-like products before?

- ☐ Yes → *Complete the rest of this section*
- ☐ No → *Skip ahead to next section*
  ("Stressors, Triggers for Tobacco Use and/or Use of Tobacco-like Products, and Barriers to Cessation")

How many times have you tried to quit? ______________

What is the longest period of time you quit for? ________

When was your last quit attempt? ____________________

This toolbox may not be reproduced or distributed, in whole or in part, without prior permission. For more information, please contact the NPLSC lead, Nardine Nakhla, at nnakhla@uwaterloo.ca

If the following cessation therapies were used, indicate:

	Efficacy	Number of Weeks Used	Reason(s) for Stopping
Short-acting NRT (*e.g., gum, lozenges, inhaler, spray*)			
Long-acting NRT (*e.g., patches*)			
Bupropion SR (Zyban®)			
Varenicline (Champix®)			
E-Cigarettes			
Acupuncture			
Herbal remedies			
Hypnosis			
Laser			
Other (*specify*):			

What did you learn from previous attempts to reduce or quit?

What led you to resume use of tobacco and/or tobacco-like products?

- ☐ Discharge from healthcare site
- ☐ Withdrawal symptoms
- ☐ Stopped medication
- ☐ Stopped behavioural support
- ☐ Use of alcohol or other drugs
- ☐ Negative mood
- ☐ Habit
- ☐ Being with other smokers
- ☐ Stress
- ☐ Other (*specify*): ____________________

Stressors, Triggers for Tobacco Use and/or Use of Tobacco-like Products, and Barriers to Cessation

What are stressors in your life?

- ☐ Financial
- ☐ Work or unemployment
- ☐ Family
- ☐ Mental health issues
- ☐ Physical illness
- ☐ Housing
- ☐ Other (*specify*): ____________________

This toolbox may not be reproduced or distributed, in whole or in part, without prior permission. For more information, please contact the NPLSC lead, Nardine Nakhla, at nnakhla@uwaterloo.ca

What are your triggers for tobacco use and/or use of tobacco-like products?

- ☐ Other smokers in the home
- ☐ Dealing with stress
- ☐ Work
- ☐ Social events
- ☐ Other (*specify*): ____________________

What do you like about smoking or using tobacco/tobacco-like products?

What don't you like about smoking or using tobacco/tobacco-like products?

Why do you want to quit using tobacco and/or tobacco-like products?

- ☐ Health
- ☐ Cost
- ☐ Social pressure
- ☐ Family reasons
- ☐ Other (*specify*): ____________________

Barriers or concerns about reducing or stopping:

- ☐ Withdrawal symptoms
- ☐ Cravings
- ☐ Stress/stress relief
- ☐ Enjoyment
- ☐ Boredom
- ☐ Depression
- ☐ Weight gain
- ☐ Discouragement/lack of willpower
- ☐ Fear of failure
- ☐ Work environment
- ☐ Home environment
- ☐ Cost of medication
- ☐ Cost/timing of groups
- ☐ Disruption of social relations
- ☐ Loss of time to self/breaks
- ☐ Other (*specify*): ____________________

This toolbox may not be reproduced or distributed, in whole or in part, without prior permission. For more information, please contact the NPLSC lead, Nardine Nakhla, at nnakhla@uwaterloo.ca

Does your drug benefit plan cover cessation medications?

- ☐ Yes
- ☐ No
- ☐ Don’t know
- ☐ Not applicable - no benefit plan

Next Steps

- ☐ Proceed with pharmacist-led management for cessation
  - ☐ *Optional – set a quit date (dd/mm/yy):* ______________________________
- ☐ Refer to primary care provider for further assessment/management

Rationale:

Name of pharmacist: ______________________________

Registration number: ______________________________

Date: ______________________________

Pharmacy name: ______________________________

Phone number: ______________________________

Fax number: ______________________________

This toolbox may not be reproduced or distributed, in whole or in part, without prior permission. For more information, please contact the NPLSC lead, Nardine Nakhla, at nnakhla@uwaterloo.ca

TREATMENT

Considering pharmacotherapy?

Yes → **1st Line Pharmacotherapy Options[a]**

No → *Quitting, with or without pharmacotherapy, has been associated with the emergence of serious neuropsychiatric symptoms*

MONITOR CLOSELY

REMINDER:
Pharmacotherapy increases the odds of quitting and should be offered to all tobacco users without contraindications to drug therapy

Long-acting + short-acting NRT[b]

Does the patient have any of the following contraindications to therapy?

Gum

- ☐ Dentures, partial, or crown
- ☐ TMJ

Patch

- ☐ Allergy to adhesive

If yes → consider alternative therapy and/or refer to primary care provider

No → ☐ Initiate NRT
(according to cigarettes/day[c];)
+ Non-pharm strategies

Varenicline (*Champix*®)

Does the patient have any of the following contraindications to therapy?

- ☐ Pregnant or planning pregnancy
- ☐ Breastfeeding
- ☐ <18 years of age
- ☐ History of renal failure & taking cimetidine
- ☐ Previous reaction to varenicline

If yes → consider alternative therapy and/or refer to primary care provider

No → ☐ Initiate varenicline
(8-35 days before quit date)
Days 1-3: 0.5 mg qAM
Days 4–7: 0.5 mg BID
Day 8-12 weeks:[d] 0.5-1 mg BID
+ Non-pharm strategies

Bupropion SR (*Zyban*®)

Does the patient have any of the following contraindications to therapy?

- ☐ History of seizure disorder or head trauma
- ☐ Currently taking bupropion (e.g., Zyban or Wellbutrin)
- ☐ Previous reaction to bupropion
- ☐ Current or previous eating disorder
- ☐ Severe hepatic impairment
- ☐ Excessive use of alcohol/sedatives
- ☐ Use of MAO inhibitor within the past 14 days

If yes → consider alternative therapy and/or refer to primary care provider

No → ☐ Initiate bupropion SR
(8 days before quit date)
Days 1-3: 150 mg qAM
Day 4-12 weeks:[e] 150 mg BID
+ Non-pharm strategies

Non-pharmacological strategies

- ☐ Drink water
- ☐ Use distraction techniques and/or delay tactics
- ☐ Remove tobacco from environment
- ☐ Avoid triggers
- ☐ Use relaxation techniques or meditation
- ☐ Join a tobacco cessation support group
- ☐ Start an exercise program

Note: list not exhaustive

	<10	10-19	20-29	30-39	40+
Patch strength	☐ 7 mg	☐ 14 mg	☐ 21 mg	☐ 28 mg *(21 mg + 7 mg)*	☐ 42 mg *(21 mg x2)*
If time to 1st cigarette is <30 mins after waking, consider higher patch strength, as indicated	☐ 14 mg	☐ 21 mg	☐ 28 mg *(21 mg + 7 mg)*	☐ 35 mg *(21 mg + 14 mg)*	☐ ______________
	**PLUS**		**PLUS**		
Short-acting NRT	☐ Spray<br>☐ Inhaler<br>☐ 2 mg lozenge<br>☐ 2 mg gum		☐ Spray<br>☐ Inhaler<br>☐ 4 mg lozenge<br>☐ 4 mg gum		

If 1st line options are ineffective or inappropriate, consider 2nd or 3rd line options, respectively *(see page 14)*

[a]Choice should be based on: 1) evidence for efficacy, 2) clinical suitability *(e.g., contraindications, drug interactions)*, 3) patient preference & options previously tried
[b]Consider NRT prior to other pharmacologic options
[c]Consult additional resources for other tobacco types
[d]0.5 mg BID may be indicated in patients with CrCl <30 mL/min, those who experience adverse effects at higher doses, and elderly patients
[e]Dose adjustment to 150 mg daily may be necessary for patients on oral hypoglycemics or insulin, those with hepatic or renal insufficiency, and elderly patients

This toolbox may not be reproduced or distributed, in whole or in part, without prior permission. For more information, please contact the NPLSC lead, Nardine Nakhla, at nnakhla@uwaterloo.ca

2nd Line Pharmacotherapy Options

Varenicline (*Champix®*) + NRT | **Bupropion SR (*Zyban®*) + NRT** | **Varenicline (*Champix®*) + Bupropion SR (*Zyban®*)**

Does the patient have contraindications to therapy (*see page 13*)?
If yes → consider alternative therapy and/or refer to primary care provider

No ↓

☐ Initiate varenicline (*Champix®*) + NRT

Varenicline: same dosing as 1st line (*see page 1*)
NRT: different doses over 12 weeks

+ Non-pharm strategies

No ↓

☐ Initiate bupropion SR (*Zyban®*) + short-acting NRT +/- NRT patch

Monitor blood pressure

+ Non-pharm strategies

No ↓

☐ Initiate varenicline (*Champix®*) + bupropion SR (*Zyban®*)

Same dosing as 1st line (*see page 1*)

+ Non-pharm strategies

If 2nd line options are ineffective or inappropriate, consider 3rd line options

3rd Line Pharmacotherapy Options

Clonidine

Does the patient have any of the following contraindications to therapy?

- ☐ Severe bradyarrhythmia
- ☐ Previous reaction to clonidine
- ☐ Galactosaemia (*depending on product*)

If yes → consider alternative therapy and/or refer to primary care provider

No ↓

☐ Initiate clonidine

PO: 0.1 mg daily initially
Transdermal: 100-200 mcg/day patch q7days

+ Non-pharm strategies

Nortriptyline

Does the patient have any of the following contraindications to therapy?

- ☐ Acute recovery phase of MI
- ☐ Previous reaction to nortriptyline
- ☐ Use of MAO inhibitor within the past 14 days
- ☐ Use of linezolid or IV methylene blue

If yes → consider alternative therapy and/or refer to primary care provider

No ↓

☐ Initiate nortriptyline (*10-28 days before quit date*)

Initial: 25 mg daily
Target: 75-100 mg daily for ≥12 weeks post-quit date

+ Non-pharm strategies

Cytisine (*Cravv®*)

Does the patient have any of the following contraindications to therapy?

- ☐ Pregnant or planning pregnancy
- ☐ Breastfeeding
- ☐ Advanced atherosclerosis
- ☐ Uncontrolled hypertension

If yes → consider alternative therapy and/or refer to primary care provider

No ↓

☐ Initiate cytisine (*Cravv®*)

Day 1-3: 1 cap 6x daily q2h with a corresponding reduction in # of cigarettes smoked for the 1st 3 days
Day 4-12: 1 cap q2.5 h (*5 caps daily*)
Day 13-16: 1 cap q3 h (*4 caps daily*)
Day 17-20: 1 cap q5 h (*3 caps daily*)
Day 21-25: 1-2 caps daily
Note: *smoking cessation should occur by day 5*

+ Non-pharm strategies

REMINDER:
Drugs metabolized by 1A2 or 2E1 may need dose adjustment with smoking cessation
(e.g., caffeine should be ↓ by 50%)
For a complete list of drugs that interact with tobacco smoke, see:
https://smokingcessationleadership.ucsf.edu/factsheets/drug-interactions-tobacco-smoke-rx-change-2019

References
1. University of Ottawa Heart Institute. Ottawa model for smoking cessation in primary care: quit plan consult form. Updated April 2018.
2. Pharmacists for a Smoke-Free Canada, Ontario Pharmacists Association. Canadian pharmacist smoking cessation pharmacotherapy algorithm. Published December 21, 2018.
3. Selby, P. Tobacco use disorder: smoking cessation. In: Compendium of Therapeutic Choices. Ottawa, ON: Canadian Pharmacists Association. [Updated May 2018; Accessed November 12, 2019]. https://myrxtx.ca.
4. zpharm, Inc. Cravv®: frequently asked questions. https://www.zpharm.ca/faqs/. Accessed November 12, 2019.
5. Clonidine. In: Lexi-Drugs. Hudson, OH: Lexi-Comp, Inc. [Updated November 11, 2019; Accessed November 12, 2019]. http://online.lexi.com.proxy.lib.uwaterloo.ca/lco/action/home.
6. Nortriptyline. In: Lexi-Drugs. Hudson, OH: Lexi-Comp, Inc. [Updated November 12, 2019; Accessed November 12, 2019]. http://online.lexi.com.proxy.lib.uwaterloo.ca/lco/action/home

This toolbox may not be reproduced or distributed, in whole or in part, without prior permission. For more information, please contact the NPLSC lead, Nardine Nakhla, at nnakhla@uwaterloo.ca

OTHER APPROACHES FOR MANAGEMENT

1 MOTIVATIONAL INTERVIEWING

Explore the **5R's** using reflective listening:

**R**elevance	Why is quitting relevant to health, family, etc.?
**R**ewards	What are the potential benefits of quitting?
**R**isk	What are the acute and chronic risks of tobacco use?
**R**oadblocks	What are roadblocks to quitting?
**R**epetition	Repeat motivational interviewing during every encounter with patient

2 REDUCE TO QUIT

Step 1
(*0-6 weeks*)

- Set target # of cigarettes/day* to cut down by (*usually at least 50%*) and a date to achieve this
- Use short-acting NRT to manage cravings

Step 2
(*6 weeks-6 months*)

- Continue to cut down cigarettes* using NRT
- Goal should be complete cessation by 6 months

Step 3
(*within 9 months*)

- Stop all cigarettes* and continue NRT to relieve cravings

Step 4
(*within 12 months*)

- Cut down amount of NRT used, then stop NRT completely
- ***Note:*** *some patients may require NRT for longer periods - less harmful than continuing to smoke*

*Consult additional resources for other tobacco types

This toolbox may not be reproduced or distributed, in whole or in part, without prior permission. For more information, please contact the NPLSC lead, Nardine Nakhla, at nnakhla@uwaterloo.ca

PRESCRIBING DOCUMENTATION

(for use where applicable)

Name: ______________________

Date of birth: ______________________

Health card number (*or equivalent*):

____

Gender (*if required/relevant*): ______________________

Address: ______________________

____

Telephone number: ______________________

Date: ______________________

Medication & strength: ______________________

Directions: ______________________

Quantity: ______________________

Refills: ______________________

Rationale for prescribing, including desired or expected outcome(s) (*e.g., cessation vs. reduction*):

Prescribing pharmacist: ______________________

Signature: ______________________

Registration #: ______________________

Pharmacy name: ______________________

Address: ______________________

____

Phone number: ______________________

Fax number: ______________________

Primary care provider notified?

☐ No → *Rationale:* ______________________

☐ Yes → Name of primary care provider notified: ______________________

Title: ______________________

Method of notification

☐ *Fax (#:* ______________________*)*

☐ *Other:* ______________________

Date sent: ______________________

This toolbox may not be reproduced or distributed, in whole or in part, without prior permission. For more information, please contact the NPLSC lead, Nardine Nakhla, at nnakhla@uwaterloo.ca
